# Medical Fees in Cottage Hospitals

**Published:** 1899-04-22

**Authors:** 


### EDITOR'S LETTER-BOX.

[Our Correspondents are reminded that prolixity is a great bar to publication, and that brevity of style and conciseness of statement greatly facilitate early insertion.]

### MEDICAL FEES IN COTTAGE HOSPITALS.

"Treasurer" writes: I would beg a little space in your columns to elicit some information on a question of cottage hospital management. At the institution in which I am interested we have five medical officers—being the doctors of the town—who attend on a rota, and are supposed to give gratuitous services. This is theory—practice is different. We have never had a case of a patient being attended by a doctor as medical officer. Patients of the poorer classes are recommended, by their doctors already attending, to obtain admission; they get a subscriber's ticket, and the doctor continues his visits (allowed by our rules), sees his patient through, and sends in his bill to him. The committee may or may not, according to the patient's circumstances, make a charge for board, nursing, &c. Is this system of the doctors making charges to cottage hospital patients usual? It must be understood that the committee, as such, know nothing of these charges. They are, or should be, an arrangement between the doctor and the patient before the latter's admission. It does not seem reasonable that a mechanic, for instance, who would naturally pay his doctor if he were attended in his own home, should escape this charge simply because he has received the further benefit of skilled nursing and careful treatment which our wards supply. At the same time there is a general idea that what a doctor does in hospital should be done gratuitously. I should be glad to know whether there are places where a similar custom obtains, and also what *quid pro quo* a medical man gets if he foregoes these charges. If he be debarred from charging I should be afraid he would cease to recommend and use the hospital, to the detriment of the charity and the loss of the deserving poor.

